# The prevalence of lower eyelid epiblepharon and its association with refractive errors in Chinese preschool children: a cross-sectional study

**DOI:** 10.1186/s12886-020-01749-7

**Published:** 2021-01-04

**Authors:** Deyi Zhuo, Si Chen, Xiaofang Ren, Bingsong Wang, Linbo Liu, Lin Xiao

**Affiliations:** 1 Department of Ophthalmology, Beijing Shijitan Hospital, Capital University of Medical Sciences, Beijing, 100143 People’s Republic of China; 2 Department of Ophthalmology, Chinese PLA General Hospital, Beijing, 100853 People’s Republic of China; 3 School of Electrical and Electronic Engineering, Nanyang Technological University, Singapore, 639798 Singapore; 4 Department of Ophthalmology, Beijing Tongren Hospital, Capital University of Medical Sciences, Beijing, 100730 People’s Republic of China; 5 School of Chemical and Biomedical Engineering, Nanyang Technological University, Singapore, 637459 Singapore

**Keywords:** Lower eyelid epiblepharon, Astigmatism, Myopia, Refractive error, Preschool children

## Abstract

**Background:**

To assess the prevalence and demographics of lower eyelid epiblepharon in Chinese preschool children and to evaluate its association with refractive errors.

**Methods:**

In this population-based, cross-sectional study, a total of 3170 children aged 3 to 6 years from Beijing, China underwent examinations including weight, height, cycloplegic autorefraction and slit-lamp examination of external eyes. The prevalence of lower eyelid epiblepharon in preschool children was evaluated and its association with age, sex, body mass index (BMI), and refractive errors was analyzed using logistic regression analysis.

**Results:**

The prevalence of lower eyelid epiblepharon was 26.2%, which decreased with age, with prevalence in 3-, 4-, 5-, and 6-year-olds of 30.6, 28.0, 15.0, and 14.3%, respectively. Boys had a higher risk of having epiblepharon than girls (OR = 1.41; 95%CI, (1.20–1.66)) and no significant correlation was detected between BMI and epiblepharon after adjusting for age and sex (*p* = 0.062). Epiblepharon was significantly associated with a higher risk of refractive errors, including astigmatism (OR = 3.41; 95% CI, (2.68–4.33)), myopia (OR = 3.55; 95% CI, (1.86–6.76)), and hyperopia (OR = 1.53; 95% CI, (1.18–1.99)).

**Conclusions:**

There is a high prevalence of lower eyelid epiblepharon in Chinese preschool children, particularly among boys and younger children. Preschoolers with lower eyelid epiblepharon are subject to a higher risk of developing astigmatism, myopia, and hyperopia, than those without. Increased attention should be paid to this eyelid abnormality in the preschool population.

## Background

Epiblepharon is an eyelid disorder characterized by a horizontal skinfold that can overlap the eyelid margin, which results in eyelash brushing against the ocular surface [1, 2]. This eyelid anomaly usually affects the lower eyelid bilaterally and is reported to be common among East Asian descendants [3]. Although it more commonly involves no or mild symptoms, there are still considerable numbers of patients subject to epiblepharon-related discomforts such as tearing, irritation, and photophobia combined with keratopathy given the ever-increasing Asian populations worldwide [4–6].

Several researchers have attempted to document its prevalence and demographic or clinical characteristics in Asian populations including but not limited to Japanese [3, 7], Korean [8–10], and Chinese populations [4, 5, 11, 12]. However, to the best of our knowledge, its prevalence in Chinese preschool children is currently unknown. In addition, although the correlation between epiblepharon and refractive errors particularly astigmatism has been previously evaluated [4, 5, 8–12], this association has yet to be assessed in the general population since previous researchers retrospectively reviewed clinical data exclusively from epiblepharon patients who sought medical or surgical treatment at local hospitals [4, 5, 8–12].

In this study, we conducted a population-based study with the aim of exploring the prevalence and demographic characteristics of lower eyelid epiblepharon in Chinese preschool children and assessing its correlation with refractive errors including astigmatism, myopia, and hyperopia.

## Methods

### Subjects

This was a cross-sectional, school-based study of lower eyelid epiblepharon conducted from July to September 2017 in Xicheng District, a district with approximately 20,000 kindergartners in Beijing. Two-stage stratified cluster sampling was used to select students for study inclusion. In the first stage, 20 kindergartens were randomly selected from 120 kindergartens in Xicheng District. In the second stage, all preschoolers from the 20 kindergartens were selected to undergo health examination at Xicheng Maternal and Child Health Care Hospital. The sample size was calculated to be 3458 with a prevalence rate of 10%, a 1% error rate and a 95% confidence interval based on the study performed by Noda S [3]. A total of 3721 children aged 3–6 years participated in the study and 3170 of them were included in the data analysis, yielding a completion rate of 85.2%. All participants and their parents or guardians were given full knowledge of the study and written informed consent was obtained from at least one of their parents or guardians.

### Physical and eye examinations

All examinations were conducted at room temperature (~ 26–28 °C). The height of the children was measured in meters without shoes, and the weight was measured in kilograms. BMI was calculated as weight in kilograms divided by the square of height in meters.

All participants underwent external-eye examination of both eyes by the same ophthalmologist (ZD) using a slit-lamp biomicroscope. Photographs of both eyes were taken using a digital camera against a white background for further diagnosis and classification of epiblepharon. Refractive error was determined by cycloplegic refraction performed with a handheld autorefractor (SureSight® Autorefractor, Welch Allyn) ≥ 30 mins after cycloplegia which was induced by 3 drops of 1% cyclopentolate (Cyclogyl, Alcon, Belgium) with an interval of 10 mins. For quality control, the autorefractor was calibrated every day prior to data collection and approximately 5% of the children were randomly selected to repeat the refraction test.

### Definitions of lower eyelid epiblepharon

**Fig. 1:**
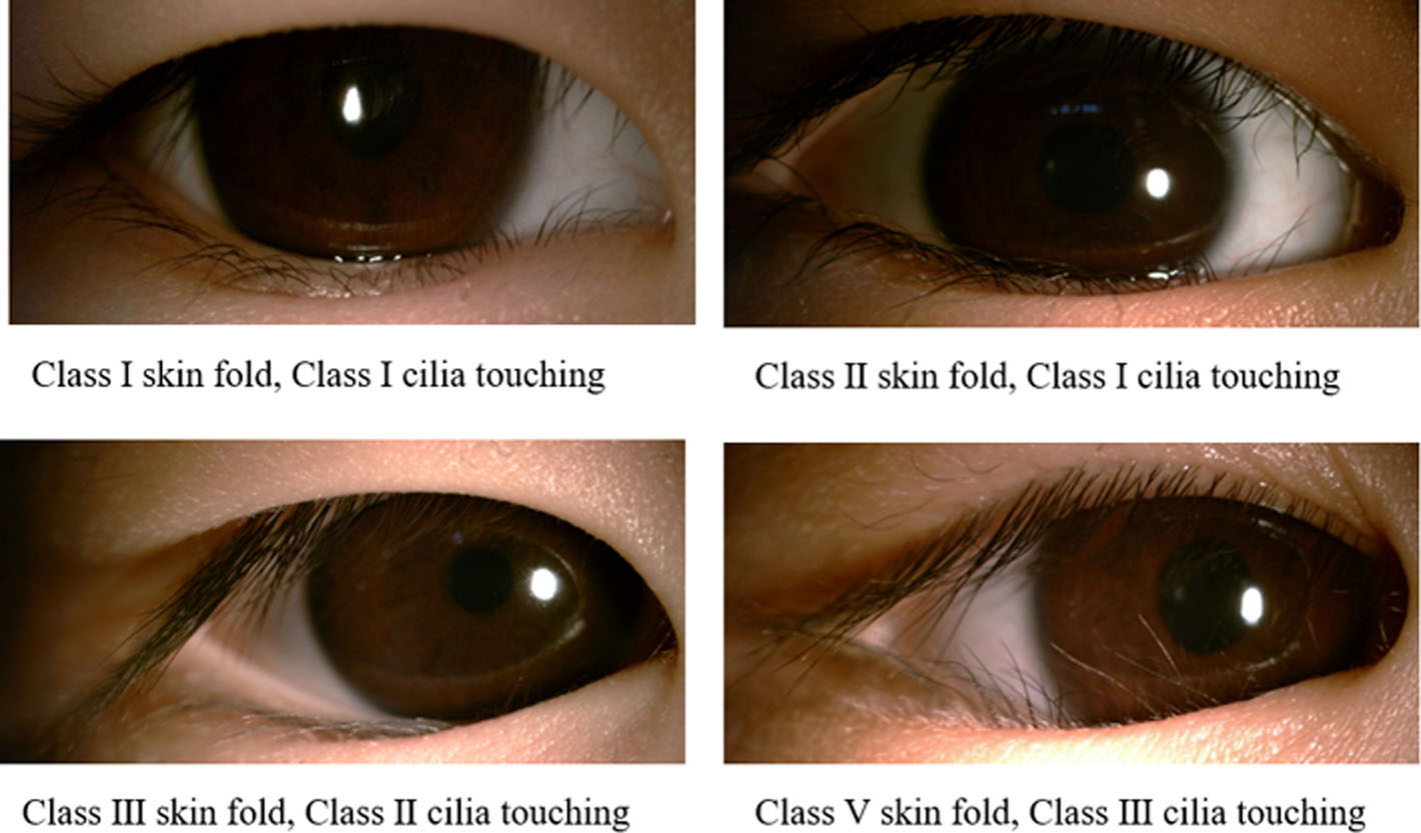
Classification of lower eyelid epiblepharon

Lower eyelid epiblepharon is diagnosed as a redundant skinfold in the lower eyelid with inverted eyelashes touching the corneal surface, but no inward rotation of the eyelid margin [3]. According to Khwarg’s classification, the severity of skinfold was categorized into 4 classes according to its height and the degree to which it concealed the eyelid margin in the primary eye position [8]; the severity of cilia-cornea touch was classified into 3 classes according to the area of inverted cilia touching the ocular surface in the primary position (Fig. 1) [8].The children were thereafter considered to have mild epiblepharon if they had any signs of class I, moderate epiblepharon if they demonstrated any signs of class II, and severe epiblepharon if they presented any signs of class III or worse [13]. The diagnosis of lower eyelid epiblepharon was performed independently by two authors (ZD and CS) where disagreement was resolved by discussion with a senior specialist (XL).

### Definitions of myopia, hyperopia and astigmatism

The spherical equivalent (SE) refractive error was expressed as sphere power + ½ × cylinder power. Myopia was defined as SE ≤ − 0.50 diopter (D), and hyperopia was defined as SE ≥ + 2.00 D. Emmetropia was defined as SE between − 0.50 D and + 1.00 D, non-inclusive. Astigmatism was defined as a cylindrical refractive error (CYL) of at least 1.50 D with cylindrical refractive error presented as negative correcting cylinder form and was classified into three categories, i.e., with-the-rule astigmatism (WTR; cylinder axis between 1° and 15° or 165° and 180°), against-the-rule astigmatism (ATR; cylinder axis between 75° to 105°), and oblique astigmatism (OBL; cylinder axis between 16° and 74° or 106° and 164°). For children with lower eyelid epiblepharon, if both eyes had equal severity of this condition, refractive error of the right eye was used for analysis; otherwise, the worse eye was selected. For those without epiblepharon, if both eyes had equivalent refractive error, data from the right eye were used for analysis; otherwise, the worse eye was used.

### Statistical analysis

All the data were imported to Epi-Info version 7.0.9.7 for double-entry customizing, and any differences were resolved by checking with the original data. Chi-square test was employed to analyze the differences in sex, age, and refractive errors between children with and without lower eyelid epiblepharon. Independent samples t-tests were used to compare the differences in height, weight, and BMI between children with and without lower eyelid epiblepharon. One-way analysis of variance (ANOVA) was used to assess the differences in the cylindrical power among astigmatic children with different severities of epiblepharon. Multivariable logistic regression analysis was used to evaluate the factors associated with lower eyelid epiblepharon, and odds ratios (ORs) and the 95% confidence intervals (CIs) were calculated. All analyses were performed using SPSS (version 22, SPSS Inc., Chicago, Illinois), and a two-sided *p* < 0.05 was considered to be statistically significant.

## Results

There were 1652 boys and 1518 girls aged 3–6 years included in the analysis and 551 children were excluded owing to incompletion of the study (*n* = 548), true entropion (*n* = 1) and external hordeolum (*n* = 2). There was no significant difference in age between boys (4.10 ± 0.75 yrs) and girls (4.08 ± 0.76 yrs) of all included subjects (independent samples t-test; *p* = 0.46). Overall, 26.2% (830 out of 3170) children had lower eyelid epiblepharon, which could be further grouped into mild (54.3%), moderate (29.6%) and severe (16.1%) classes based on the severity of skin-fold height and cilia-corneal touch (Table 1).

**Table 1:** Classification of lower eyelid epiblepharon

Class of cilia-cornea touching	Class of skin fold height				Total
	I	II	III	IV	
**I**	451 (54.3%)^a^	84 (10.1%)^b^	1 (0.1%)^c^	0 (0%)^c^	536 (64.5%)
**II**	2 (0.2%)^b^	160 (19.3%)^b^	66 (8.0%)^c^	1 (0.1%)^c^	229 (27.6%)
**III**	0 (0%)^c^	3 (0.5%)^c^	36 (4.3%)^c^	26 (3.1%)^c^	65 (7.9%)
**Total**	453 (54.5%)	247 (29.9%)	103 (12.4%)	27 (3.2%)	830 (100%)

^a^Mild epiblepharon

^b^Moderate epiblepharon

^c^Severe epiblepharon

Table 2 summarizes the general profile of all included children. Statistically significant differences were detected in sex, age, SE, CYL, astigmatism type (chi-square tests; *p* = < 0.001, < 0.001, < 0.001, < 0.001, and 0.022, respectively) and BMI (independent samples t-test; *p* = 0.018) between epiblepharon and nonepiblepharon children (Table 2); no significant differences were identified in either height or weight between these two groups (independent samples t-test; *p* = 0.303 and 0.413, respectively) (Table 2).

**Table 2:** Characteristics of children with and without lower eyelid epiblepharon

	Epiblepharon (***n*** = 830)	No epiblepharon (***n*** = 2340)	***P*** value
**Gender, n (%)**			< 0.001*
**Boys**	487 (29.5)	1165 (70.5)	
**Girls**	343 (22.6)	1175 (77.4)	
**Age (yrs), n (%)**			< 0.001*
**3**	152 (30.6)	344 (69.4)	
**4**	601 (28.0)	1547 (72.0)	
**5**	39 (15.0)	221 (85.0)	
**6**	38 (14.3)	228 (85.7)	
**SE (D), n (%)**			< 0.001*
**≤ − 0.5**	22 (56.4)	17 (43.6)	
**- 0.5 to 2**	713 (25.0)	2141 (75.0)	
**≥ 2**	95 (34.3)	182 (65.7)	
**CYL (D), n (%)**			< 0.001*
**0 to <1**	541 (22.3)	1890 (77.7)	
**1 to <1.5**	132 (30.6)	300 (69.4)	
**1.5 to <2**	68 (43.0)	90 (57.0)	
**2 to < 2.5**	44 (57.1)	33 (42.9)	
**2.5 to < 3**	32 (65.3)	17 (34.7)	
**> 3.0**	13 (56.5)	10 (43.5)	
**Astigmatism type, n (%)**			0.022*
**WTR**	127 (52.3)	112 (47.4)	
**ATR**	7 (35.5)	20 (64.5)	
**OBL**	23 (56.1)	18 (43.9)	
**Height (ms), mean (SD)**	1.01 (0.07)	1.01 (0.07)	0.303
**Weight (kgs), mean (SD)**	15.72 (2.92)	15.63 (2.73)	0.413
**BMI, mean (SD)**	15.43 (1.83)	15.26 (1.69)	0.018*

*BMI* Body mass index, *SD* Standard deviation, *CI* Confidence interval, *SE* Spherical equivalent refractive error, *CYL* Cylindrical refractive error, *D* Diopters, *WTR* With-the-rule, *ATR* Against-the-rule, *OBL* Oblique

*p is statistically significant at 5%

**Fig. 2:**
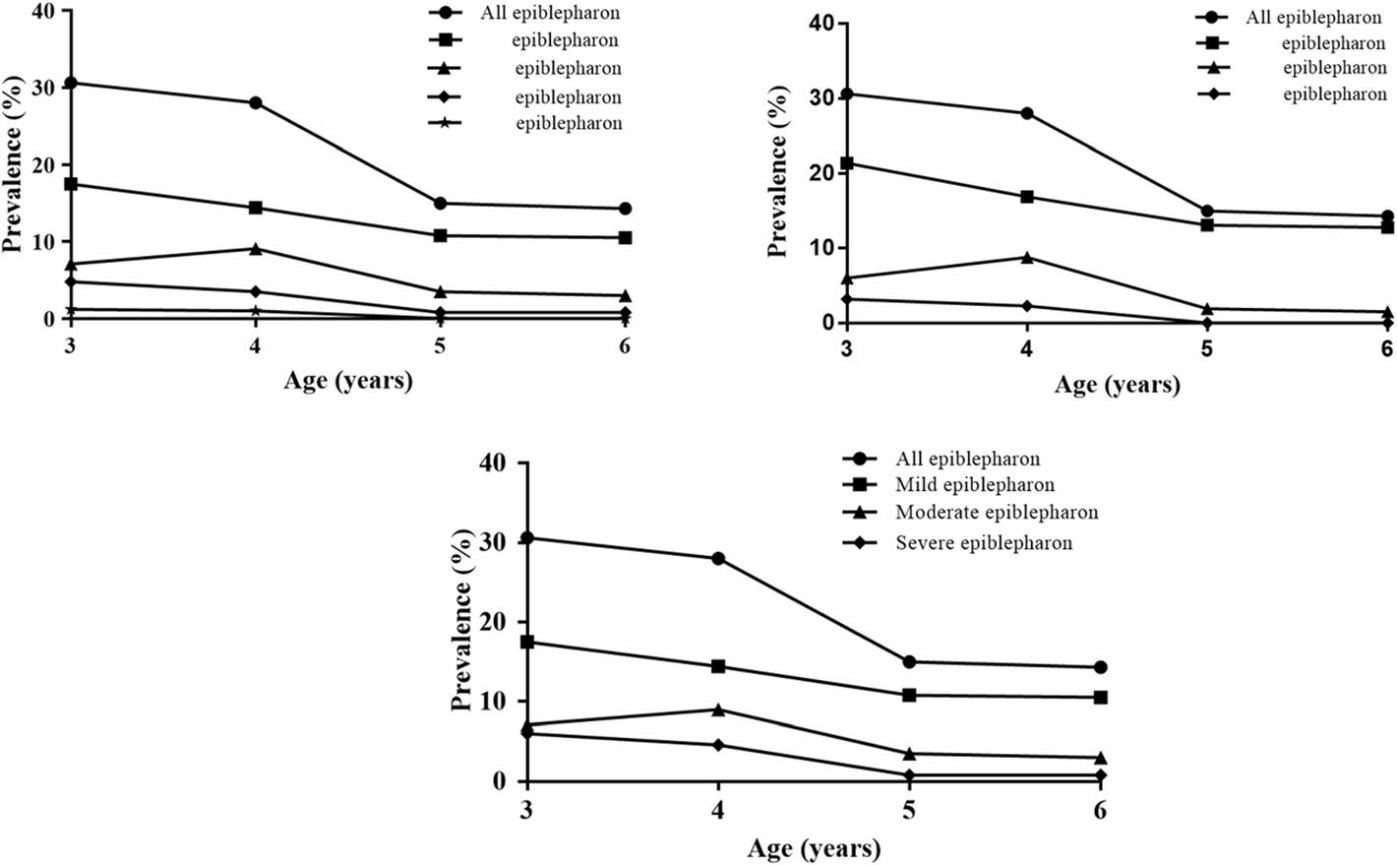
Age-specific prevalence of lower eyelid epiblepharon by its severity. Epiblepharon classified by skinfold height (**a**), cilia-cornea touching area (**b**), and both skinfold height and cilia-cornea touching area (**c**)

The prevalence of lower eyelid epiblepharon based on age was 30.6, 28.0, 15.0, and 14.3% for 3-, 4-, 5-, and 6-year-old children, respectively (Table 2). Figure 2 further illustrates the age-specific prevalence of lower eyelid epiblepharon by its severity according to criteria based on skin-fold height only (Fig. 2a), cilia-cornea touching area only (Fig. 2b), and criteria established by both (Fig. 2c). At different degrees of severity, younger patients roughly demonstrated a higher prevalence of epiblepharon than older patients (Fig. 2).

### Lower eyelid epiblepharon associated risk factors

As statistically significant differences in age, sex and BMI were detected between children with and without lower eyelid epiblepharon (Table 2), multivariable logistic regression analysis was performed to further evaluate the association between lower eyelid epiblepharon and these three variables to screen for the risk factors for this eyelid disorder (Table 3). After adjustment for potential confounders including age, sex and BMI, epiblepharon was significantly associated with sex and age. Specifically, boys were more likely to have epiblepharon than girls (OR = 1.41 with *p* < 0.001); younger children at 3, 4, and 5 years old demonstrated higher possibilities of having epiblepharon than older children (6 years old), although no statistically significant difference was identified between 5- and 6-year-old children (OR = 3.68, 2.95, and 1.24 with *p* = < 0.001, < 0.001 and 0.402, respectively). However, after adjusting for sex and age, the association between BMI and epiblepharon was no longer statistically significant (*p* = 0.062).

**Table 3:** Association between lower eyelid epiblepharon and age, gender, and BMI

Risk factors	OR	95%CI	***P*** value
**Gender**			
**Boys vs girls**	1.41	(1.20, 1.66)	*P* < 0.001
**Age (yrs)**			
**3 vs 6**	3.68	(2.38, 5.68)	*p* < 0.001
**4 vs 6**	2.95	(2.01, 4.32)	*p* < 0.001
**5 vs 6**	1.24	(0.75, 2.03)	*p* = 0.402
**BMI**	1.07	(0.95, 1.15)	*p* = 0.062

*OR* Odds ratio, *CI* Confidential interval

### Relation between lower eyelid epiblepharon and refractive errors

Astigmatism (CYL ≥ 1.5 D) was identified in 307 children (9.68%) and 157 of them had epiblepharon. A statistically significantly higher prevalence of astigmatism was detected in epiblepharon children than in nonepiblepharon children (18.9% vs 6.4%) (chi-square test; *p* < 0.001). Meanwhile, in both populations, the astigmatism type was largely WTR (80.9% for epiblepharon vs 74.7% for nonepiblepharon).

Myopia (SE ≤ − 0.5 D) was recognized in 39 children (1.23%) with 22 of them having epiblepharon; hyperopia (SE ≥ 2 D) was found in 277 children (8.74%) with 95 of them being epiblepharon-positive. Higher prevalences of myopia (2.7% vs 0.7%) and hyperopia (11.4% vs 7.8%) were identified in epiblepharon children than in nonepiblepharon children (chi-square tests; *p* = < 0.01 and 0.002, respectively). To further evaluate the association between lower eyelid epiblepharon and refractive errors, multivariable logistic regression analysis was performed where age and sex were adjusted for (Table 4). Overall, epiblepharon children presented an increased risk of astigmatism relative to nonepiblepharon children (OR = 3.41; 95% CI, (2.68, 4.33)), and epiblepharon preschoolers had a higher risk of myopia (OR = 3.55; 95% CI, (1.86, 6.76)) and hyperopia (OR = 1.53 95% CI, (1.18, 1.99)) than nonepiblepharon children.

**Table 4:** Association between lower eyelid epiblepharon and refractive errors

	Astigmatism(≥1.5D)		Myopia(≤ − 0.5D)		Hyperopia(≥2.0D)	
	OR (95%CI)	*P* value	OR (95%CI)	*P* value	OR (95%CI)	*P* value
**No Epiblepharon**	1.00 (ref)		1.00 (ref)		1.00 (ref)	
**Epiblepharon**						
**Mild**	3.15 (2.35, 4.22)	< 0.001	4.38 (2.14, 8.95)	< 0.001	1.51 (1.09, 2.10)	0.014
**Moderate**	3.82 (2.69, 5.42)	< 0.001	4.00 (1.64, 9.75)	0.002	1.52 (1.00, 2.32)	0.050
**Severe**	3.55 (2.24, 5.62)	< 0.001	–	–	1.62 (0.94, 2.79)	0.082
**All**	3.41 (2.68, 4.33)	< 0.001	3.55 (1.86, 6.76)	< 0.001	1.53 (1.18, 1.99)	0.001

*OR* Odd ratio, *WTR* With the rule, *OBL* Oblique

## Discussion

This population-based cross-sectional study investigated the prevalence and demographics of lower eyelid epiblepharon and explored its correlation with refractive errors in 3- to 6-year-old Chinese children. To the best of our knowledge, our study is the first to reveal the prevalence of this eyelid disorder in Chinese preschoolers and analyzes the relationship between epiblepharon and refractive errors including not only astigmatism but also myopia and hyperopia. We observed a prevalence of 26.2% in 3- to 6-year-old Chinese children, much higher than that of Noda S. et al.’s study in Japanese children, where 13.8% of 766 children were diagnosed with epiblepharon [3]. The relatively lower prevalence in Noda’s study is possibly because they adopt relatively stricter criteria for disease diagnosis where the widely accepted Khwarg’s classification has yet to be established. In Noda’s study, cilia-cornea touching was confirmed only when the cornea was positively stained after topical fluorescein staining, whereas later studies by Khwang S. et al. and Young S. et al. indicated that a considerable number of epiblepharon patients (usually those with mild epiblepharon) may not have the ocular manifestation of corneal erosion that could be stained with fluorescein [8, 13]. In addition, when we excluded children with mild epiblepharon, the prevalence decreased to 11.9%, which was similar to the 13.8% reported in Noda’s study.

Given such a high prevalence in Asian infants and children, deducing the demographic characteristics of epiblepharon is helpful for a better understanding of this eyelid disorder. To date, limited data are available from either observations of the general population [3, 7] or retrospective data from patients who underwent surgical treatment [4]. Consistent with previous studies [3, 4, 7], our results in Chinese preschool children show that younger individuals have a higher risk of epiblepharon, supporting the widely-accepted hypothesis that epiblepharon tends to disappear spontaneously with aging as facial bone growth in Asians [6]. However, there are discrepancies in BMI and the sexual predilection between our study and others. Different from results in Japanese and Singaporeans where no sexual predilection was recognized [3, 4, 7], the present study demonstrates that boys are subject to a higher risk of having lower eyelid epiblepharon than girls with an adjusted OR = 1.41 in the logistic regression model. Differences in the diagnostic criteria [3], subjects’ age range [7], epiblepharon severity, and possible sample selection bias in a clinic-based study [4] may all contribute to the discrepancy in sex predilection between our study and others.

Although lower eyelid epiblepharon trends to resolve spontaneously with aging, its occurrence during the critical period of visual development makes it crucial to disclose if it is correlated with refractive errors, which may ultimately lead to permanent visual impairment such as amblyopia or retinopathy [14–17]. In this study, the prevalence of astigmatism was 9.68%, similar to previous studies in Asian infants and preschoolers [18–21], and, in those with lower eyelid epiblepharon, the prevalence could be as high as 18.9%. We demonstrate for the first time that epiblepharon children are subject to a significantly higher risk of astigmatism, largely being WTR in the general population, and these findings agree well with previous retrospective studies in patients [9, 11, 12]. In addition, we also evaluated the relationship between lower eyelid epiblepharon and spherical refractive errors, i.e., myopia and hyperopia. These epiblepharon preschoolers were 3.55 times as likely to have myopia as those without epiblepharon, and 1.53 times as likely to have hyperopia. The significantly increased risk of myopia in epiblepharon children agrees with the observations in clinical patients that severe myopia is commonly accompanied by a large number of cilia touching the cornea [9]. However, considering the relatively small sample size (22 out of 39 epiblepharon preschoolers being myopic) and absence of severe myopia (− 2.75 D to − 0.5 D), further study with a larger sample size should be conducted to further validate the association between epiblepharon and myopia.

The strength of this study lies in the fact that the demographics of epiblepharon and its association with refractive errors are evaluated in the Chinese preschool population with by far the largest sample size thus far reported. Nevertheless, there are several limitations in this study. First, the children included in this study were all enrolled from urban areas, which may result in bias from sample selection. Further studies ideally from multiple centers including children from both rural and urban area would improve our understanding of the risk factors related to lower eyelid epiblepharon and its association with refractive errors. Second, the prevalence of epiblepharon may be underestimated because epiblepharon can be missed in children whose cilia touch the cornea only in downward gaze but not in primary gaze. Another limitation is that we used only the skinfold height and the cilia-cornea touching area as criteria for the diagnosis of lower eyelid epiblepharon without considering areas of corneal erosion. However, owing to concerns about the risk of the fluorescein dying procedure and limited time allowance in a screening circumstance, an approach that is both safe and simple must be adopted; in addition, this limitation can be mitigated since there is high agreement among the three criteria for the diagnosis of epiblepharon [8]. In addition, our conclusion may be affected by type 1 error as multiple comparisons were conducted.

## Conclusions

our study demonstrates a relatively high prevalence of lower eyelid epiblepharon in Chinese preschoolers, particularly in boys and young children, and shows that there are significant correlations between lower eyelid epiblepharon and refractive errors, including astigmatism, myopia, and hyperopia. Given such a high prevalence combined with the increased risk for refractive errors, it would be of great significance to be aware of the necessity and importance to establish an effective screening strategy for this disease, to conduct a closer follow-up of the clinical manifestations of involved children and to consider giving early interventions and visual rehabilitation when warranted.

## Data Availability

The datasets used and/or analyzed during the current study are available from the corresponding author on reasonable request.

## Abbreviations

AORs: Adjusted odds ratios
ORs: Odds ratios
CIs: Confidence intervals
BMI: Body mass index
SD: Standard deviation
SE: Spherical equivalent refractive error
CYL: Cylindrical refractive error
WTR: With-the-rule
ATR: Against-the-rule
OBL: Oblique
